# The Profession in England

**Published:** 1860-01

**Authors:** 


*The Profession in England.*—Two schools of instruction for dental students are now in operation in England—one under the patronage of the Odontological Society and the other under that of the College of Dentists. This substantial proof of progress is most agreeable intelligence in this country; but, on the other hand, we are sorry to learn that internecine controversies are raging with great virulence. The differences between the Odontological Society and the College of Dentists, leading to crimination and recrimination of the most unpleasant character, of which the evidences are to be found in the recent numbers of the English dental periodicals, are greatly to be deplored as delaying the legitimate advance of the profession. Another source of trouble exists in the somewhat doubtful position of the profession in relation to the other branches of medicine. The medical commission, under the late "medical act," have brought suit against several persons for the use or abuse of the word *surgeon* in the title "Dental Surgeon," or "Surgeon Dentist," and it has been determined that, as *surgeon* implies some desire or ability on the part of the surgeon dentist to practice in some measure as surgeons, thereby deceiving the public, no dentist is to be permitted to use that word unless duly authorized by the College of Surgeons and registered according to the provisions of the medical act. Since these cases have occurred, the legislature has made provision for the dentist, ranking him after practitioners of midwifery. It is to be hoped that some measures will soon be taken by which an institution may be founded for the purpose of giving dental instruction, with the ability to confer a legal degree, entirely independent of any other specialty, on the English dental student. In this country the juridicial machinery of medicine in all of its branches is much more simple and satisfactory than in England. With one degree, which is an assurance to the public of so much professional instruction, we are killed or cured. We die as contentedly under the hands of a full doctor as we would under the feeble excuse that might be urged if our attendant were only licentiate or M. R. C. S.; and when we are cured, which is an event, we apprehend, as proportionably frequent in this country as any other, we are as grateful as the nature of the case requires. So our doctor of dental surgery is as much and as legally a doctor as if he were to receive degrees from every college in Europe. It has been apparently impossible for our English friends, with that ludicrous air of superiority which is so common, to appreciate the fact that we can give degrees in any thing. This by the way, and as an illustration, we recently had occasion to observe the studious care with which a medical man of this country was eased of his title in some remarks appended to an article which he had communicated to a scientific journal of London. This was the more striking as the letters M. D. were plainly visible at the head of the article.

